# The use of systematic reviews in the planning, design and conduct of randomised trials: a retrospective cohort of NIHR HTA funded trials

**DOI:** 10.1186/1745-6215-14-S1-O92

**Published:** 2013-11-29

**Authors:** Ashley Jones, Elizabeth Conroy, Paula Williamson, Mike Clarke, Carrol Gamble

**Affiliations:** 1 University of Liverpool, Liverpool, UK; 2 Queens University, Belfast, UK

## Background

There has been limited research on how systematic reviews are used within the design of new trials. Here we report an investigation of how systematic reviews have informed the planning and design of a cohort of randomised trials.

## Methods

Documentation from the application process for all randomised trials funded by the National Institute for Health Research Health Technology Assessment (NIHR HTA) programme between 2006 and 2008 were obtained. Data were extracted on references to systematic reviews and how reviews had been used in the planning and design of the trial.

## Results

Documentation was available for 48 of the 50 randomised trials funded by NIHR HTA during this period. The main areas in which systematic reviews were used were in the selection or definition of an outcome to be measured in the trial (7 of 37, 19%), the sample size calculation (7, 19%), the duration of follow up (8, 22%) and the approach to describing adverse events (9, 24%).

## Conclusions

Systematic reviews were referenced in most funded applications, with just over half having used the review to inform the design. Although there is an expectation from funders that applicants will use a systematic review to justify the need for a new trial, there seems to be little expectation regarding further use of a review in the planning and design of the trial. Guidelines for applicants and funders should be developed to promote the use of systematic reviews in the planning and design of randomised trials.

